# Childhood Hypertrophic Cardiomyopathy: A Disease of the Cardiac Sarcomere

**DOI:** 10.3389/fped.2021.708679

**Published:** 2021-07-02

**Authors:** Gabrielle Norrish, Ella Field, Juan P. Kaski

**Affiliations:** ^1^Centre for Inherited Cardiovascular Diseases, Great Ormond Street Hospital, London, United Kingdom; ^2^Institute of Cardiovascular Sciences University College London, London, United Kingdom

**Keywords:** paediatric, hypertrophic cardiomyopathy, sarcomere, sudden death, progression

## Abstract

Hypertrophic cardiomyopathy is the second most common cause of cardiomyopathy presenting during childhood and whilst its underlying aetiology is variable, the majority of disease is caused by sarcomeric protein gene variants. Sarcomeric disease can present at any age with highly variable disease phenotype, progression and outcomes. The majority have good childhood-outcomes with reported 5-year survival rates above 80%. However, childhood onset disease is associated with considerable life-long morbidity and mortality, including a higher SCD rate during childhood than seen in adults. Management is currently focused on relieving symptoms and preventing disease-related complications, but the possibility of future disease-modifying therapies offers an exciting opportunity to modulate disease expression and outcomes in these young patients.

The prevalence of childhood Hypertrophic Cardiomyopathy (HCM) is estimated to be ~3 in 100,000 (1) live births with an annual incidence between 0.24 and 0.47/100,000 (1–3), making it the second most common cardiomyopathy presenting during childhood. The underlying aetiology is more heterogeneous than seen in adult populations and includes inborn errors of metabolism, RASopathy syndromes and neuromuscular disease. Historically, it was believed that sarcomeric disease was uncommon during childhood but it is now recognised that in most children, HCM is caused by sarcomere protein gene variants. This article provides a review of current knowledge of sarcomeric childhood HCM, highlighting the variability in disease expression and unanswered questions.

## Genetics of Sarcomeric HCM in Childhood

Up to two thirds of children with non-syndromic HCM have a disease-causing variant in a sarcomere protein gene identified on genetic testing (4–6), which is usually inherited as an autosomal dominant trait. A sarcomeric variant is less likely to be identified in those presenting in infancy (approximately one third) but the overall yield of genetic testing in childhood is similar to, or even higher than, reported in adult cohorts (7). The majority of variants (70–80%) are found in β-myosin heavy chain (MYH7) or myosin-binding protein C (MYBPC3) with the proportion of disease attributable to MYBPC3 increasing with age. A smaller number of patients have variants in other sarcomeric proteins (4, 6, 8) as described in Table 1. Homozygosity or compound heterozygosity has been associated with early onset disease, severe phenotypes and poor clinical outcomes but is uncommon (<5%) even in childhood cohorts (18, 19, 21). The majority of childhood onset disease is therefore caused by single disease-causing sarcomeric variants. Small paediatric studies have reported that predictors of finding a disease-causing variant in childhood patients include a family history of HCM, pattern and degree of hypertrophy (22).

**Table 1:** Reported genotype-phenotype correlations in hypertrophic cardiomyopathy.

**Disease causing variant**	**%**	**Gene**	**Reported genotype-phenotype associations**
Thick filament	75–80%	Myosin Binding Protein C3(MYBPC3)	Disease expression heterogeneous, marked age related penetrance (late onset disease common). (9, 10) Founder mutations seen (11).
		Myosin Heavy Chain 7 (MYH7)	Variants in the converter region of MYH7 associated with worse prognosis (12). Restrictive phenotypes reported (13, 14)
Thin filament	15–20%	Troponin T (TNNT2)	Minimal hypertrophy (15). Right atrial dilatation (13). Small studies reported an increased arrhythmic risk but this has not been replicated (15, 16).
		Troponin I (TNNI3)	Restrictive phenotype (14)
		Alpha-cardiac actin (ACTC)	Apical hypertrophy and LVNC (17)
		Essential myosin light chain (MYL3), Regulatory myosin light chain (MYL2), Alpha-tropomyosin (TPM1), Cardiac troponin C (TNNC1), Alpha myosin heavy chain (MYH6)	
Other	<1	*Z* disk proteins (e.g., CSPR3), phospholamban	
Compound heterozygosity/homozygosity	<5%	MYBPC3	Early onset, severe hypertrophy and poor outcome (18–20)

Familial sarcomeric HCM is associated with age-related and variable penetrance (23). Whilst some individuals will develop hypertrophy in early childhood, other mutation carriers may never develop significant features of disease (23). Disease phenotype, progression, and outcomes are likewise highly variable, even amongst family members carrying an identical mutation (9). Childhood-onset disease is more likely if there is a family history of early-onset disease (24) but as yet unidentified genetic and epigenetic modifiers are likely to play an important role in the expression of primary disease-causing sarcomeric mutations. Recent genome wide association studies have described the importance of common genetic variants on the risk of developing a HCM phenotype although the contribution of additional variants to childhood phenotypes has not yet been described (25, 26).

## Presentations and Symptoms

The underlying aetiology is at least partly responsible for determining the age of presentation with childhood HCM and patients with underlying metabolic or syndromic disease are more likely to present in infancy or early childhood (27–29). While sarcomeric HCM has historically been described to be a disease of adolescence or early adulthood, recent European and North American population cohort studies have shown a peak in presentation during adolescence and also describe presentation throughout the childhood years, including an important minority (~14%) in the first year of life (6, 29). Such studies highlight that sarcomeric disease can present at any age, including the very young.

A diagnosis of HCM in childhood may be made following referral for symptoms (such as chest pain, palpitations, syncope, or dyspnoea), ECG abnormalities or through family screening (24, 27, 30, 31). A small proportion (3–4%) are diagnosed following a resuscitated cardiac arrest (1, 32). Infants have been described as being more likely to present with symptoms, but this may reflect the inclusion of patients with syndromic disease in population studies who are recognised to have a higher prevalence of left ventricular systolic impairment and heart failure symptoms at presentation (27, 33).

## Disease Phenotype

### Pre-phenotypic Features

**Figure 1:**
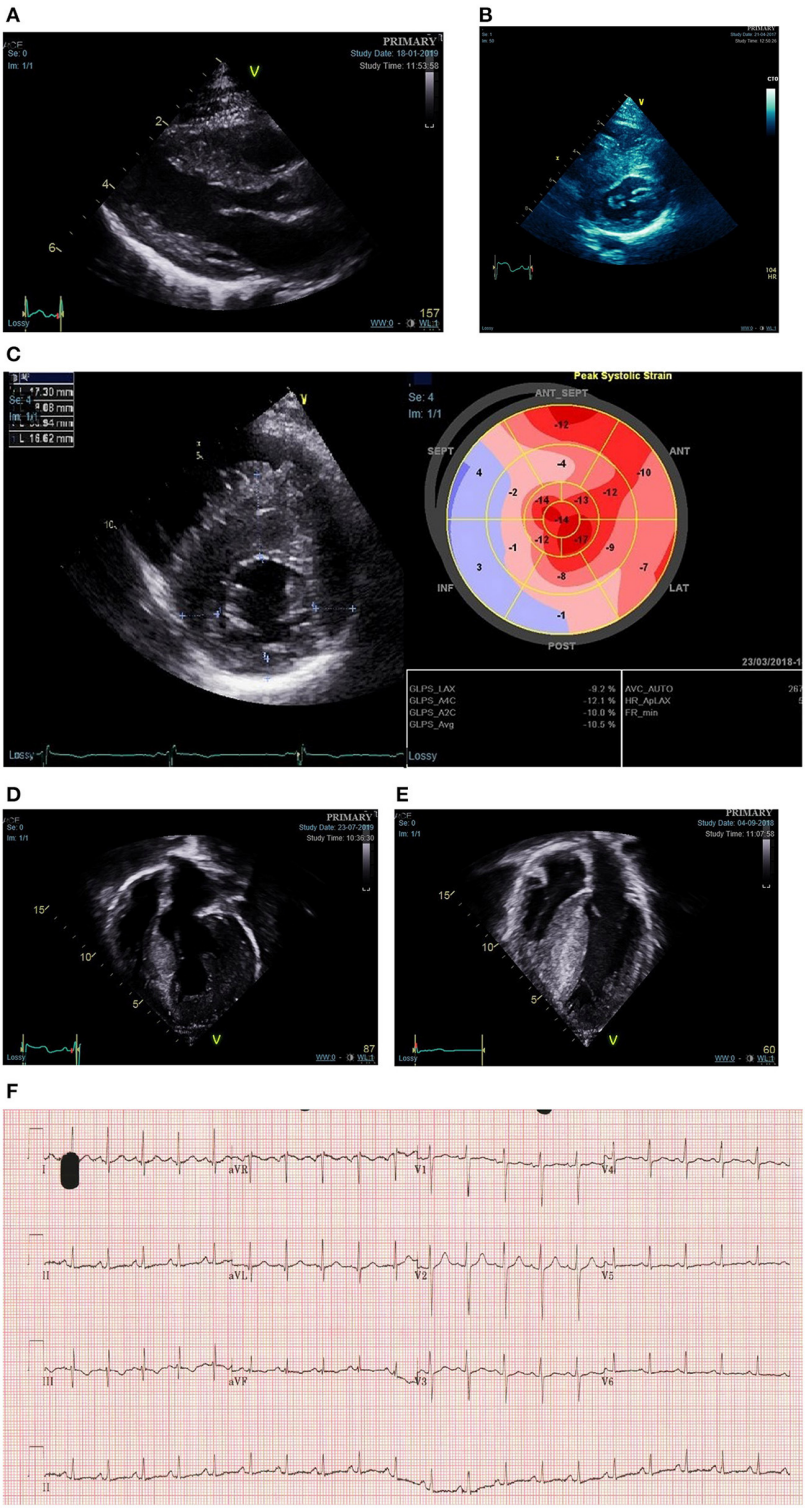
Phenotypic spectrum of sarcomeric hypertrophic cardiomyopathy presenting during childhood. **(A)** Mild asymmetric LVH secondary to a disease causing MYH7 variant diagnosed in infancy through family screening. **(B)** Moderate-severe asymmetric LVH secondary to disease causing MYBPC3 variant diagnosed in infancy following referral with murmur. **(C)** Reduced longitudinal strain in a teenager with asymmetric LVH and familial disease. **(D)** Severe eccentric hypertrophy in a 10-year old with a disease causing alpha-tropomyosin variant. **(E)** Biventricular hypertrophy in a teenager with compound MYBPC3 variants. **(F)** ECG showing abnormal repolarisation (flat or negative T waves infero-laterally) in a child heterozygous for familial MYH7 variant with no LVH on echocardiogram.

Predictive genetic testing as part of family screening has led to the identification of a growing population of genotype-positive phenotype-negative individuals. Although these individuals have not been shown to be at risk of disease-related complications such as life threatening malignant arrhythmias (23, 34, 35), they require ongoing clinical screening to detect phenotype development. Previous studies have described early “pre-phenotypic” features of sarcomere mutation carriers including ECG abnormalities (23, 24, 34), impaired LV relaxation (36), elongated mitral valve leaflets (37), or myocardial crypts (36–38) (Figure 1). Recent studies have identified independent predictors of developing a phenotype during follow up, including male sex or an abnormal ECG (23). However, our understanding of how these abnormalities relate to, and predict, the development of LV hypertrophy in childhood remains incomplete.

### Phenotype at Diagnosis During Childhood

Left ventricular hypertrophy (LVH), as defined by a maximal left ventricular wall thickness >2 standard deviations (≥2 *Z*-score) above body surface area (BSA) corrected mean, is a pre-requisite for the diagnosis of childhood HCM (39, 40). The interpretation of all *z*-score thresholds is hampered by the use of different normative data for *z*-score calculations, each of which yields different *z*-scores for the same individual. It has, however, been suggested that the current BSA-corrected threshold for diagnosis in childhood is too low and should be increased to *Z* ≥ 6 in line with the diagnostic threshold in adults of MLVWT ≥15 mm (or ≥13 mm for familial disease) (40). Sarcomeric HCM is typically characterised by asymmetric septal hypertrophy, although other morphologies of LVH (concentric, eccentric, or biventricular) more commonly associated with syndromic disease can also be seen in sarcomeric HCM (27–29).

The current paradigm, that the development of LVH in sarcomeric disease is uncommon during childhood, was based on a small study of 39 patients with familial disease in which increases in hypertrophy were seen more frequently in adolescence (41). However, studies have now shown that LVH can develop at any age in familial disease and indeed that the majority of childhood disease diagnosed through family screening met diagnostic criteria during pre-adolescence, with a significant proportion diagnosed in infancy (24, 31). One implication of the traditional paradigm is that younger children, if diagnosed, have phenotypically mild disease. More recent data from large, retrospective cohort studies have provided novel insights into the phenotype of childhood sarcomeric disease which challenge these assumptions. The HCM Risk-Kids and PRIMaCY cohorts included 1,072 and 572 patients, respectively, with non-syndromic childhood HCM, respectively, from international cardiac centres (42, 43). In both cohorts, the severity of hypertrophy was highly variable, and included a significant proportion with extreme hypertrophy (13% MLVWT ≥20 in HCM Risk-Kids). Importantly, the degree of hypertrophy was not dependent on age. Although diagnosis in infancy has traditionally been attributed to rare presentation of severe disease, recent studies have reported conflicting phenotypic findings in this population. In a UK cohort, MLVWT *Z*-score was similar for those presenting in infancy and later childhood (29), whilst recent data from the largely North American SHaRE registry reported a higher MLVWT *Z*-score for those presenting under the age of 1 (6). Together, these data suggest that the pattern and degree of hypertrophy in childhood sarcomeric disease is highly variable and can include both severe and mild LVH at any age (Figure 1).

Although LVH is a pre-requisite for diagnosis, additional morphologic abnormalities contribute to the overall phenotype of an individual patient. Left ventricular outflow tract (LVOT) obstruction [defined as a maximal LVOT gradient, as measured using Doppler echocardiography, above 30 mm (39)] is reported to be present in 22–60% (32, 44, 45) reflecting the heterogeneous nature of disease and differing definitions in published cohorts. LVOT obstruction (LVOTO) has been reported to be more common in those presenting in infancy (6). The mechanism for LVOTO is primarily systolic anterior motion of the mitral valve (SAM), with additional contributing factors in some cases including an anatomically narrowed LVOT, fixed LVOT obstruction and accessory mitral valve chords. Children with sarcomeric HCM are less likely to have complex LVOT obstruction due to a polyvalvulopathy with abnormal chordal attachments of the mitral valve as is seen in RASopathy syndromes (46). Global measures of LV systolic function (e.g., ejection fraction) are typically hyperdynamic; reduced ejection fraction is very rare in sarcomeric childhood disease and should prompt clinicians to search for underlying metabolic or syndromic disease (47, 48). However, myocardial deformation imaging may identify regional subclinical myocardial dysfunction, typically located in the area of maximal hypertrophy and longitudinal axis function is often impaired even in those with hyperdynamic systolic function (49, 50) (Figure 1). In contrast, diastolic dysfunction is commonly seen in established childhood disease and may even precede the development of hypertrophy (36). Data from the HCM-Risk Kids cohort shows that phenotypic features of severe disease (severe hypertrophy, LVOT obstruction, left atrial dilatation) often co-exist and that such patients are more likely to be symptomatic (42).

### Disease Progression During Childhood

Childhood is a time of significant somatic growth and the cardiac phenotype of sarcomeric childhood HCM is highly variable and rapidly changing. However, our understanding of disease progression during childhood remains incomplete. Studies describing the follow up of childhood relatives (24, 31) or genotype-positive children (36, 51), have demonstrated that, in familial disease, increases in absolute and body-surface area corrected MLVWT occur throughout childhood. In a large single-centre cohort of over 1,000 paediatric first-degree relatives, screening appeared to identify two distinct populations (24). A small, but important, group of patients diagnosed before adolescent years with an accelerated progression of LVH, and a second, larger, group of patients diagnosed in later adolescence. In a minority of adult sarcomeric HCM patients (≈5%) evolution to a dilated hypokinetic phase with LV dilatation, systolic dysfunction and LV wall thinning has been described (52). Although this is the most common indication for heart transplantation in children with HCM (53), this is exceedingly rare in childhood-onset disease. However, several patients in the previously described familial screening cohort appeared to reach peak MLVWT during childhood, which could suggest that early onset disease may be associated with accelerated progression to end-stage disease (24). Future studies exploring the progression of sarcomeric childhood-onset disease are required.

### Genotype-Phenotype Correlations

The presence of any disease-causing sarcomeric variant has been associated with earlier disease onset and more severe LVH (54, 55). However, efforts to explore genotype-phenotype correlations in sarcomeric HCM have been limited by significant genetic heterogeneity and variable or incomplete and age-related penetrance. Table 1 describes reported genotype-phenotype correlations.

## Long Term Outcomes and Mortality

Early publications from small, highly selected tertiary centres reported that the long-term prognosis of childhood HCM was poor, with annual mortality rates of up to 7% (56, 57). Over time, larger, unselected population studies have reported lower mortality rates, which are more representative of the wider childhood HCM population. However, significant variability in outcomes exist and are largely dependent on age of presentation and underlying aetiology. European and North American population cohort studies have described children with “idiopathic” (presumed sarcomeric) disease to have a relatively good prognosis, with estimated 1 and 5-year survival of 94.4% (95% CI 92.5–96.4) and 82.2% (95% CI 76.2–88.2), respectively (28, 29). Infant-onset “idiopathic” disease was associated with worse prognosis (1-year mortality up to 14%) but for those that survived to 1 year after diagnosis, long-term outcomes were comparable to later-onset childhood disease (58). Heart-failure related deaths are responsible for the majority of mortality in infancy, whereas sudden cardiac death is the most common cause of death outside of infancy occurring at a rate of 1–2% per year (29, 42, 43, 58).

Whilst short term outcomes may be favourable, recent longitudinal data from the SHaRE registry have highlighted the cumulative morbidity and mortality associated with childhood-onset disease (6). Of children diagnosed between 1 and 18 years, 20% had experienced a cardiac event within 10 years of baseline assessment, which increased to 50% by 25 years of follow-up. Half of the early events were ventricular arrhythmias, whereas later events were more commonly heart failure-related or atrial fibrillation. Although end-stage disease is rarely observed during childhood, 40% had impaired LV systolic function by the age of 40 years and 20% had atrial fibrillation. This suggests that the lifelong burden of a childhood diagnosis is considerable and greater than previously appreciated. Compared to adults, children were more likely to experience malignant arrhythmias and twice as likely to require advanced heart failure treatments such as a cardiac transplantation or a left ventricular assist device. Interestingly, although infant-onset disease was associated with worse initial prognosis, for those that survived to 1 year, outcomes were better than childhood-onset disease with a lower cumulative incidence of heart failure, ventricular arrhythmias or atrial fibrillation providing further evidence that infant-onset disease is not necessarily a poor prognostic marker.

Predicting outcomes in childhood HCM is challenging because of the significant variability in age, aetiology, cardiac phenotype, and natural history. Multiple studies, in mixed childhood populations, have shown that presentation with symptoms of congestive cardiac failure is associated with higher cardiovascular mortality over follow up (28, 33, 59). Certain phenotypic features, largely associated with syndromic disease, including concentric LVH, biventricular hypertrophy, severe LVH and impaired systolic function, have also been associated with worse prognosis (27, 33, 58). Our understanding of the role that genotype plays in long-term outcomes is incomplete. The presence of a sarcomeric variant has been associated with a higher cumulative lifetime risk of experiencing an adverse cardiac event for both childhood and adult-onset HCM (54, 60, 61). However, the natural history of sarcomeric disease appears to differ by age of presentation with genotype positive children at increased risk of heart failure event whilst genotype positive adults are at increased risk of atrial fibrillation or all-cause mortality (6). A detailed discussion of the risk factors for arrhythmic events in childhood sarcomeric HCM can be found below but future studies exploring risk factors that predict mortality in childhood onset childhood HCM are required.

## Management of Childhood HCM

The management of children with sarcomeric HCM focuses on three main areas; family screening and counselling of family members, management of symptoms, and preventing disease-related complications. A proposed follow up schedule is shown in Table 2.

**Table 2:** Proposed follow up and investigations for childhood Hypertrophic cardiomyopathy.

**Time frame**	**Clinical review and investigations**
6–12 months	Clinical review of symptoms, Transthoracic echocardiogram, 12 lead ECG Risk stratification of arrhythmic events
12–24 months	24-h ambulatory ECG—surveillance for malignant arrhythmias and to inform risk stratification
2–3 years	Cardiopulmonary exercise test (>7 years)—functional capacity assessment and arrhythmia provocation Cardiac Magnetic Resonance Imaging (CMRI) (>7 years)—LGE for fibrosis
At any time during follow up	Genetic testing
Additional investigations if indicated	Exercise stress echocardiography—investigation for latent LVOT obstruction in symptomatic patients

## Family Screening

Disease-causing variants in sarcomere protein genes are inherited in an autosomal dominant fashion and clinical or genetic screening is recommended for all first-degree relatives (39, 40). Although clinical screening was previously recommended to commence at the age of 10 or 12 years, in light of recent evidence from family screening cohorts described above, the 2020 AHA/ACC guidelines now endorse performing clinical screening at any age following the diagnosis of HCM in a first degree relative (40). Repeat clinical assessment is required throughout childhood into adulthood due to variable and age-related penetrance, with recent estimates suggesting that up to 50% of phenotype-negative mutation carriers may develop a phenotype over 15 years (23). If a disease-causing variant has been identified in the family, at-risk family members can be offered predictive genetic testing offering the possibility of discharge from clinical follow up if they are found to be genotype negative.

## Symptom Management

Up to 70% of children with sarcomeric-HCM report cardiac symptoms, most commonly exertional or atypical chest pain, dyspnoea, palpitation or syncope (62). Chest pain is often multifactorial and can be caused by LVOT obstruction, increased wall stress due to elevated diastolic pressures leading to myocardial ischaemia, or microvascular abnormalities. Although systolic compression of epicardial and intramural vessels (myocardial bridging) is commonly seen, this is not usually of clinical importance (63). Heart failure symptoms are usually attributable to diastolic dysfunction with impaired filling. Syncope is commonly reported and has a variety of underlying mechanisms, including haemodynamic (vasovagal, LVOT obstruction or diastolic dysfunction) or arrhythmic. Investigation of the cause is important as unexplained syncope, presumed secondary to ventricular arrhythmias, is a risk factor for sudden cardiac death (42, 43, 64, 65). An understanding of the likely mechanism of symptoms is important to guide medical management.

### Symptoms in the Presence of LVOT Obstruction

Management of LVOT obstruction is typically focused on relieving symptoms. Of note, echocardiographic findings of LVOT obstruction do not correlate well with symptom severity. Beta-blockers are considered to be the first line medical therapy and are well-tolerated (66). Alternative pharmacological therapies include disopyramide (67) and calcium channel blockers (68, 69), either alone or in combination. For those with refractory symptoms or fixed obstruction, surgical myectomy has been shown to be effective at both reducing the measured gradient and providing symptomatic relief during childhood with low operative morbidity or mortality in experienced centres (70, 71). A trend toward a higher incidence of re-operation in those undergoing myectomy during infancy has been reported (72). Other invasive gradient reduction therapies, such as alcohol septal ablation of radiofrequency ablation, remain experimental in childhood and are not recommended, as the long-term effects of such therapies are unknown (73). Treatment of asymptomatic LVOTO is controversial due to conflicting reports regarding its effect on long-term prognosis. Beta-blocker therapy may be initiated in young patients who are seemingly asymptomatic, reflecting difficulties in assessing symptomatology in this patient group.

### Symptoms in the Absence of LVOT Obstruction

Symptoms in non-obstructive disease are usually secondary to impaired diastolic function or myocardial ischaemia caused by increased LV mass. Exercise echocardiography can be a useful investigation if symptoms are suggestive of LVOT obstruction to elicit latent obstructive disease (74), which has been reported in up to two thirds of children with sarcomeric disease. Treatment is largely empirical and includes beta blockers and calcium channel blockers.

## SCD Prevention

HCM is characterised by a pro-arrhythmic triad of macroscopic and microscopic features including myocyte disarray, fibrosis and small vessel disease. Sudden cardiac death is the most common cause of death, outside of infancy, in non-syndromic childhood HCM and occurs more frequently than in adult cohorts (1–2 vs. 0.8%/year) (42, 43, 75). Identifying patients at highest risk of malignant arrhythmias is therefore a cornerstone of patient management. No medical therapy is currently recommended as preventative therapy for SCD in HCM. High dose beta-blockade (up to 6 mg/kg daily) has been described to reduce the risk of arrhythmic events in a small single centre study (66). However, these results have not been confirmed independently in paediatric or adult populations. Implantable cardioverter defibrillators (ICDs) have been shown to be effective at terminating malignant ventricular arrhythmias in children with HCM but at the expense of a higher rate of complications and inappropriate therapies compared to adult patients (65, 76, 77). As this younger group of patients will have ongoing exposure to these risks throughout their lifetime, and no device or programming strategies have been shown to reduce these risks (76), the ability to identify which patients are most likely to benefit from prophylactic ICD implantation is particularly important.

### Risk Factors for SCD in Childhood HCM

Recent multi-centre collaborative studies [including HCM Risk-Kids (42), PRiMACY (43), and SHaRE registry (6)] have improved our understanding of the risk factors for SCD in childhood HCM. A previous meta-analysis of the published literature identified four major clinical risk factors for SCD in childhood HCM: previous ventricular fibrillation (VF) or sustained ventricular tachycardia (VT); unexplained syncope; NSVT; and extreme left ventricular hypertrophy (defined as a LV maximal wall thickness ≥30 mm or *Z* ≥ 6) (78). Of note, although there is robust evidence to support the use of family history of SCD as a risk factor in adult patients, there was insufficient evidence to support its use in childhood. Possible explanations for this include a higher prevalence of *de novo* variants in childhood disease, low proportion of sarcomeric disease in the reported studies or insufficient reporting of family linkage. LVH is recognised as a major risk factor, but the most useful measure of hypertrophy remains unclear. Extreme hypertrophy was only associated with SCD in half of studies using this measure and recent studies in both adult and childhood populations have described a non-linear relationship between MWT and risk, meaning those with the most severe hypertrophy do not necessarily have the highest risk of events (43, 79). The meta-analysis also suggested additional risk factors, such as left atrial diameter and LVOT obstruction, may modify an individuals' risk similar to adult disease. Table 3 describes the risk factors for SCD in childhood HCM described in the literature.

**Table 3:** Risk factors for sudden cardiac death in childhood HCM.

**Major risk factor**	**Clinical risk factor**	**Comment**
Major risk factors	Previous VF/VT	Pooled HR 5.4 (95% CI 3.67–7.95, *P* < 0.001). Pooled OR 5.06 (95% 2.11–12.17, *P* < 0.001)
	Unexplained syncope	Pooled HR 1.89 (0.69–5.16, *p* 0.22). Pooled OR 2.64 (1.21–5.79, *p* 0.02)
	NSVT	Pooled HR 2.13 (95% CI 1.21–3.74, *p* 0.0009). Pooled OR 2.05 (96% CI 0.98–4.28, *p* 0.06).
	Extreme LVH	Pooled HR 1.8 (95% CI 0.75–4.32, *p* 0.19). Pooled OR 1.70 (95% CI 0.85–3.40, *p* 0.13). The most useful measure of LVH for risk stratification is unknown.
Other putative risk factors	LA dilatation	Left atrial size was not included as a major risk factor in the meta-analysis but a significant association has subsequently been reported in four studies (32, 42, 43, 80).
	LVOT gradient	The definition of LVOT obstruction varies in the literature. Increasing LVOT gradient has been linked to SCD (32, 45) and two large studies have described an inverse relationship between LVOT gradient and risk in childhood (42, 43).
	Family history of SCD	Only 1/10 studies reported a significant association between a family history of SCD and SCD event (81). Limited evidence to support its use as a risk factor during childhood.
	Age	The role of age in SCD is not fully understood. SCD risk has been reported to be increased in pre-adolescent years (9–14 yrs) (30) and children presenting in infancy are believed to be at lower risk (27, 58)
	12 lead ECG	Proposed 12 lead ECG features include; measures of LV hypertrophy (82) and abnormal repolarisation (83). An ECG risk score has been developed by Ostman-Smith et al. (83) but this was shown to have only moderate discriminatory ability in an external validation study (84).
	LGE on CMRI	LGE has been shown to increase during childhood and is associated with left ventricular hypertrophy (51). It is unclear if LGE is an independent risk factor for SCD (85, 86).
	Genotype	The role of genotype in SCD risk during childhood is not fully understood. In small cohorts, the presence of a pathogenic sarcomeric mutation has been described to be associated with worse prognosis (61) and certain genotypes associated with higher arrhythmic risk (87).

*Adapted from Norrish et al. (78)*.

### Risk Stratification for SCD Guidelines

Little controversy exists for patients who have previously experienced a malignant ventricular arrhythmia who are widely accepted to be at high risk of future arrhythmias and are recommended for a secondary prevention ICD device (39, 40). Current risk stratification guidelines recommend the use of four major risk factors extrapolated from adult studies to identify patients for primary prevention ICD implantation: extreme LVH; unexplained syncope; NSVT; and a family history of SCD. Previous studies have shown that the co-existence of multiple risk factors has a summative effect on risk (88) and guidelines recommend a threshold for ICD implantation [≥1 risk factor in the AHA/ACC guideline (40) and ≥2 risk factors in the ESC guidelines (39)]. A validation of this approach in a cohort of childhood HCM patients from UK showed that, although the incidence of arrhythmic events increased with additional risk factors, it had a limited ability to distinguish between high and low risk patients leading to unnecessary ICD implantation in many (89).

Current practise for adult HCM patients has moved away from this approach to risk stratification, which provides relative risks for non-homogenous groups rather than individualised estimates of risk, and guidelines recommend the use of a validated risk prediction model (39, 40). HCM Risk-SCD uses readily available clinical risk factors to calculate individualised estimates of 5-year SCD risk to guide ICD implantation decisions (75). This model is not validated for use in paediatric populations but two separate paediatric risk models [HCM Risk-Kids (42) and PRiMACY (43)] have recently been published that offer clinicians the ability to calculate individualised estimates of 5-year risk of SCD using readily available clinical predictors for the first time (42, 43). The risk models differ in their age limit (16 years for HCM Risk-Kids vs. 18 years for PRiMACY), development cohort sample size (*n* = 1,072 HCM Risk-Kids vs. *n* = 572 PRiMACY) and approach to risk factor selection, which was either based on 30 years of published literature (HCM Risk-Kids) or association with the end-point on multivariable analysis in the model development cohort (PRiMACY). Nonetheless, the model predictor variables are identical (measures of LVH, left atrial diameter, NSVT, syncope), with the exception that PRiMACY also includes age as an independent predictor. These models have not yet been adopted by clinical guidelines but have been shown in independent external validation studies to have superior ability to identify patients at highest risk for arrhythmic events compared to current guidelines. The HCM-Risk-Kids model is available freely online (https://hcmriskkids.org) allowing clinicians to calculate individualised estimates of 5-year risk for their patients and perform independent external validation of the model. These models remain imperfect and future studies exploring the use of additional risk factors and serial clinical data to predict risk for childhood HCM are required.

## Disease-Modifying Treatments

To date, the management of HCM has focused on alleviating symptoms or preventing disease-related complications. However, newly developed disease-specific therapies offer an exciting opportunity to modulate disease expression. Mavacamten, a novel myosin inhibitor, has been shown in Phase III clinical trials to reduce left ventricular outflow tract gradients and improve symptoms in adults with symptomatic obstructive HCM (90). It has also been shown to cause a dose-dependent reduction in serum markers of myocardial wall stress and injury (serum natriuretic peptide and cardiac troponin) in non-obstructive adult HCM patients (91) and prevent disease expression in mouse models (92). Although studies have not yet included childhood-onset disease, such medications offer the possibility of modulating disease expression both in patients with a phenotype and genotype-positive phenotype-negative individuals identified through screening.

## Conclusion

Although the underlying aetiology of HCM presenting in childhood is heterogeneous, it is clear that it is primarily a disease of the cardiac sarcomere. Disease phenotype and progression is highly variable and includes mild and early phenotypes as well as severe disease presenting at a young age with accelerated progression to end-stage. Although overall prognosis during childhood is favourable, childhood onset disease is associated with considerable life-long morbidity and mortality, including a higher SCD rate during childhood than seen in adults. Management is currently focused on relieving symptoms and preventing disease-related complications, but the possibility of future disease-modifying therapies offers an exciting opportunity to modulate disease expression and outcomes in these young patients.

## Author Contributions

GN, EF, and JK contributed to conception and design of the review. GN and EF wrote the first draft of the manuscript. All authors contributed to manuscript revision, read, and approved the submitted version.

## Conflict of Interest

The authors declare that the research was conducted in the absence of any commercial or financial relationships that could be construed as a potential conflict of interest.
